# RELATIONSHIP OF DEMENTIA AND MEDICAID ELIGIBILITY WITH FACILITY ADMISSIONS IN MEDICARE HOME HEALTH PATIENTS

**DOI:** 10.1093/geroni/igz038.3225

**Published:** 2019-11-08

**Authors:** Jinjiao Wang, Thomas V Caprio, Helena Temkin-Greener, Xueya Cai, jingjing Shang, Yue Li

**Affiliations:** 1 University of Rochester, Rochester, New York, United States; 2 University of Rochester, Department of Public Health Sciences, NY, Rochester, New York, United States; 3 Columbia University School of Nursing, New York, New York, United States

## Abstract

This study was a secondary analysis of the Outcome and Assessment Information Set (OASIS) and administrative billing records of 6,153 adults ≥ 65 years old who received home health (HH) from a not-for-profit HH agency in upstate New York between 01/01/2017 and 12/31/2017. We examined the relationships of dementia and Medicare-Medicaid dual eligibility with unplanned institutional admission (i.e. to hospital, nursing home, or inpatient rehabilitation facility) among these HH recipients. Dementia was identified by ICD-10 codes and OASIS items (M1700, M1710, M1740). We also used OASIS record to identify dual eligible status (M0150) and unplanned facility admission (M2410 [occurrence], M0906 [date], M2430 [reason]). Time-to-facility admission was defined as the number of days from HH start date to the facility admission date. The rate of having an unplanned facility admission was 14.2% among Medicare-only patients without dementia, 15.8% among dual eligible patients without dementia, 16.7% among Medicare-only patients with dementia, and 39.3% among dual eligible patients with dementia (p<0.001). In the multivariable Cox proportional hazard model of time-to-facility admission adjusting for patient covariates, dually eligible patients with dementia were more than twice as likely as Medicare-only patients without dementia to have an unplanned facility admission (Hazard Ratio=2.35, p=0.006). This is the first study that identified synergistic effects of having both dementia and Medicare-Medicaid dual eligibility on increasing the risk of healthcare facility admission in the Medicare HH population in the United States. Policies should ensure that appropriate and sufficient HH services be provided for dually eligible patients with dementia.

